# Reconstruction of complex foot injury using anterior thigh free flap with vastus lateralis muscle pedicle and tri-cortical Iliac crest bone graft: a case report

**DOI:** 10.1097/MS9.0000000000003579

**Published:** 2025-07-16

**Authors:** Subhash Regmi, Anoj Rajkarnikar, Bishwa Bandhu Niraula, Pranodan Poudel, Paras Khakurel, Ishor Pradhan

**Affiliations:** a Department of Orthopaedics, B&B Hospital, Gwarko, Lalitpur, Nepal; b Department of Plastic Surgery, B&B Hospital, Gwarko, Lalitpur

**Keywords:** case report, chimeric flap, complex foot injury, myofasciocutaneous flap, orthoplastic, vastus lateralis muscle pedicle flap

## Abstract

**Introduction and Importance:**

Complex foot injuries from high-velocity trauma pose significant challenges due to combined bony and soft tissue damage. Early and comprehensive reconstruction using an orthoplastic approach is critical for limb salvage and achieving functional recovery.

**Presentation of Case:**

A 22-year-old male sustained a severe crush injury to the right foot following a motor vehicle accident. The injury included degloving, extensive soft tissue loss, and multiple fractures: a comminuted first metatarsal shaft fracture, a medial cuneiform bone defect, and minimally displaced fourth and fifth metatarsal fractures. Initial management included wound debridement, K-wire stabilization, and infection control. Due to dorsal skin necrosis and non-viability of the fifth toe, additional debridement and fifth ray resection were performed. A free anterolateral thigh flap with vastus lateralis muscle pedicle was used for soft tissue reconstruction. Later, a tricortical iliac crest bone graft was applied to reconstruct the medial arch and secured with titanium mini-plates and screws.

**Clinical Discussion:**

The coordinated orthoplastic approach allowed for early soft tissue coverage and delayed bony reconstruction, which are essential for functional recovery in complex foot trauma. The use of a chimeric myofasciocutaneous flap provided durable soft tissue coverage and structural support. Close multidisciplinary collaboration with Orthopaedic and Plastic Surgery team for surgical management and in-hospital management with physician, dietician and physiotherapist was vital for managing complications and optimizing outcomes.

**Conclusion:**

This case demonstrates that a multidisciplinary orthoplastic strategy with timely soft tissue and bony reconstruction can result in successful limb salvage and functional recovery in complex foot trauma. Long-term follow-up remains crucial to address any post-traumatic complications.

## Introduction

Complex foot injuries often result from high velocity trauma, such as motor vehicle accidents, and are difficult to manage because of involvement of bony and soft-tissue components^[^1^]^. These injuries often lead to significant functional disability, longer hospital stays, and high treatment costs^[^2^]^.

Some patients may also require primary amputation, resulting in poor patient satisfaction^[^3^]^. Hence, a multi-disciplinary approach, involving orthopedic surgeons, plastic surgeons, and physiotherapists is required to provide early and adequate bony and soft-tissue reconstruction to obtain optimal function^[^4,5^]^.

**HIGHLIGHTS**

- Complex foot trauma with extensive bone and soft tissue loss managed using a staged orthoplastic approach.
- Anterolateral thigh free flap with vastus lateralis muscle pedicle used for durable soft tissue coverage.
- Medial arch reconstructed using tricortical iliac crest bone graft fixed with titanium mini-plates.
- At 12-month follow-up, patient achieved full weight-bearing, American Orthopedic Foot and Ankle Society score of 80/100, and returned to work.
- Novel combination of chimeric flap and iliac crest graft showed effective functional and structural recovery.

Here, we present a case of complex foot injury, in which a combined orthoplastic approach was instituted for reconstruction. This case report has been reported in line with the SCARE 2025 checklist^[^6^]^.

### Case description

**Figure 1.**
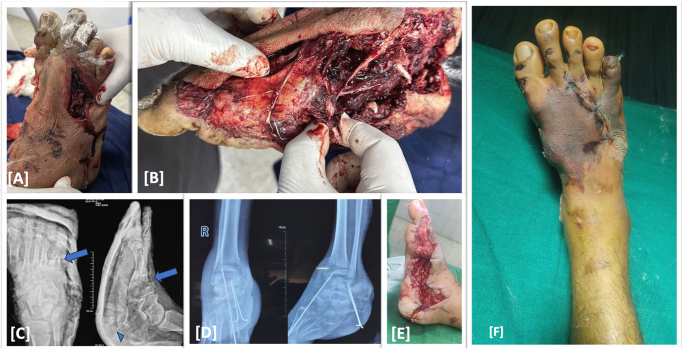
Crush injury of Right foot with lacerated skin and exposed muscle and bony fragments (A and B); Plain radiograph showing comminuted medial, middle, and lateral cuneiform fractures, 4th and 5th Meta-tarsal fractures (blue arrows), and calcaneus chip fracture(arrowhead) (C); post-operative radiograph showing K-wire stabilization (D); Post-debridement status with skin tag suturing (E); Clinical image showing evidence of infection with blackening of skin, more pronounced at 5th toe (F).

A 22-year-old male, student, non-smoker and non-alcoholic, presented to a tertiary care center following a road traffic accident, in which a motorbike rider was hit by a four-wheeler, sustaining crush injury over right foot and brought in to the emergency of Level-1 tertiary care center via ambulance 5 h after trauma at 17:00 pm, 12/11/2023. There were no other associated injuries, and he had no significant past medical and surgical history. He has no significant drug history and known allergies. Systemic examinations were normal and on local examination, there was degloving injury located at medial side of the foot with skin and soft tissue loss. Capillary refill was less than 3 s on 1st to 4th toes and 5 s on 5th toe with decreased sensation (1/2) and restricted all toes movement. The plain radiograph showed comminuted 1st metatarsal shaft fracture, medial cuneiform bone defect, minimally displaced 4th and 5th Meta-tarsal shaft fractures, and calcaneus chip fracture. Patient was initially managed with broad spectrum intravenous antibiotics, wound debridement, k-wire stabilization of metatarsal and calcaneus fracture fragments, and tag skin suturing. A long 2.0 mm K-wire was inserted intramedullary through great toe distal phalanx up to the Talus, maintaining the defect at medial cuneiform, and two 1.8 mm K-wires were placed to stabilize the calcaneal chip fracture (Fig. 1).

**Figure 2.**
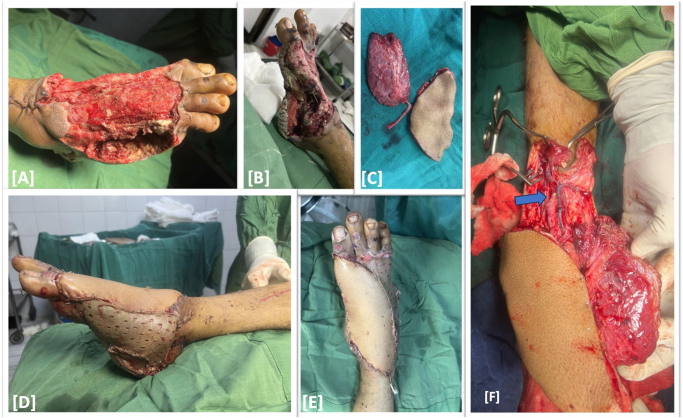
Pre-op clinical picture showing skin and soft-tissue defect at dorsal and medial aspect of the foot (A and B) and some evidence of soft tissue infection (B); Anterolateral thigh free flap with vastus lateralis muscle pedicle was harvested (C); flap was set on the defect and muscle flap was covered with split-thickness skin graft (D and E); Pedicle anastomosis (blue arrow) at the receipient site (F).

Patient underwent serial surgical debridement every 48 h. However, patient developed local infection and dorsal skin necrosis on 4th day of admission with non-viable 5th toe. Then, patient underwent 5th ray resection and thorough debridement of non-viable dorsal skin. Plastic and Reconstructive Surgery team was consulted for soft-tissue reconstruction. The defect size was approximately 22 × 18 cm^2^. On the 10th day of admission, an anterolateral thigh free flap including the muscle pedicle of the Vastus Lateralis was harvested to cover the dorsal and medial defects. The descending branch of the lateral circumflex femoral artery, along with its two venae comitantes, was used as the vascular pedicle. Both fasciocutaneous and muscular perforators arose from this descending branch, facilitating reliable flap perfusion. Split thickness skin graft was also harvested from contralateral thigh to cover muscular component and some remaining skin defects. The donor site of free flap was closed primarily. The reconstructive surgery was performed by a team of two consultant plastic surgeons with 5 years of clinical experience (Fig. 2).

**Figure 3.**
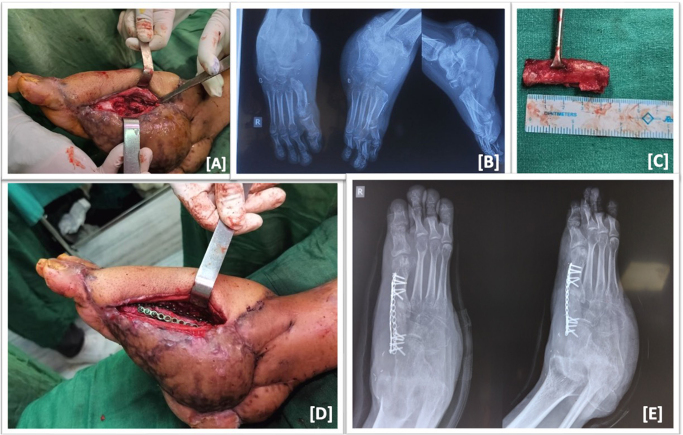
Intra-op clinical picture showing bony defect at 1st tarso-metatarsal joint (A) plain radiograph showing medial arch defect with loss of medial cuneiform (B); 4.0 cm Tri-cortical iliac crest graft was harvested (C); A slot grafting was carried out and fixed with plates and screws (D); post-operative radiographic showing adequate fixation (E).

The bony defect at medial arch, between the base of 1st meta-tarsal and navicular bone, was not addressed at the time. The post-operative period was uneventful and both flap and graft survived without any major complications. Patient was then discharged on 16th day of admission with ankle brace and non-weight bearing rehabilitation protocol. K-wires were removed at 6 weeks and ankle range of motion exercises were started. As there was enough bony stock to prevent instability and permit rehabilitation following soft tissue reconstruction, definitive bony reconstruction was delayed till there was no signs of infection and the free flap margins had matured at the defect site. Patient was then planned for 1st tarso-metatarsal joint fusion or medial arch defect reconstruction. Infection markers, complete blood count, erythrocyte sedimentation rate, and C-reactive protein level, were within normal limits. Intra-operatively, the defect size was 3.5 cm. Thus, it was reconstructed using autologous 4 cm tri-cortical iliac crest bone graft and cancellous bone grafts harvested from ipsilateral ilium. Fixation was done using two titanium mini-plates and screws. The surgery was performed by the team led by a fellowship trained orthopedic trauma surgeon with more than 5 years of experience (Fig. 3).

**Figure 4.**
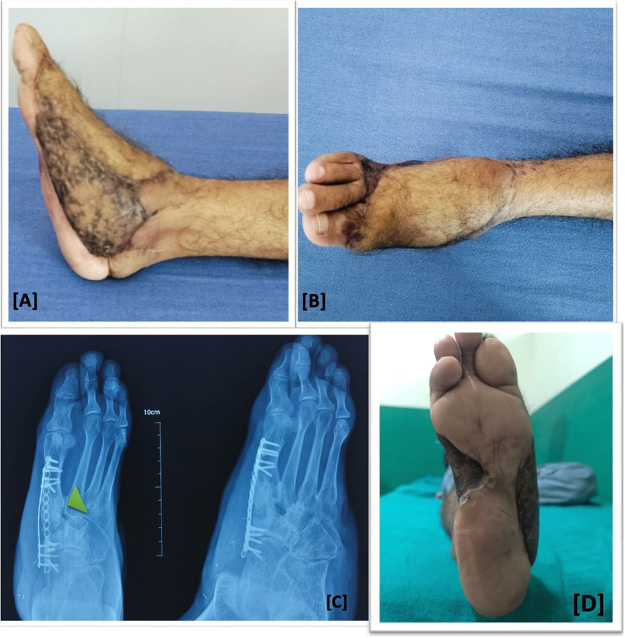
At 12-month follow-up: satisfactory aesthetic and functional recovery (A and B); plain radiograph showing complete union(arrowhead) with intact hardware (C); 17 months follow up of showing adequate normal skin at sole of foot with healed graft and flap (D).

Post-operative period was uneventful and patient was discharged on 5th post-operative day. Patient underwent home-based rehabilitation protocol advised by a senior physiotherapist with more than 10 years of experience. Patient was then followed up regularly at 6-week, 3-month, 6-month, and 12-month. Progressive weight bearing was started after 6 weeks. Patient was compliant to the rehabilitation protocol. At 18 months, patient was walking without any aid with mild, tolerable pain, and discomfort. Ankle range of motion (ROM) with 15° dorsiflexion and 30° plantarflexion. Plain radiograph showed complete union of the tri-cortical segment and some osteopenia at mid-foot. American Orthopedic Foot and Ankle Society (AOFAS) ankle and hind foot scale measurement was 80/100 (Fig. 4). The patient had a sufficient ROM in the ankle and subtalar joints, with normal ankle dorsiflexion, plantarflexion, inversion (25°) and eversion (20°) to ensure mobility. The patient demonstrated good clinical and functional outcomes; however, the procedure had some limitations. There was diminished sensation to pain, touch, temperature, and two-point discrimination over the free flap area and the skin graft site on the dorsal, medial, and lateral aspects of the foot. The plantar aspect of the foot had normal skin and without any sensory deficits. However, the patient had normal activity of daily living, was able to perform his previous job, had a plantigrade foot, and was able to mobilize without any assistive devices. He expressed satisfaction with the outcome of the procedure and reported that it had a positive impact on his quality of life and ability to return to work.

### Discussion

This report illustrated a case of complex foot injury, in which an orthoplastic approach was considered to provide early definitive treatment. These types of injuries pose significant challenge to treating surgeon because of high risk of complications, such as infection and ischemic necrosis^[^7^]^. Previous studies have shown better patient satisfaction with primary amputation rather than heroic attempt of reconstruction^[^8^]^. However, with advancement in the field of orthopedic and reconstructive surgeries and availability of tertiary care facilities, adequate reconstruction of such injuries has been the mainstay of treatment^[^9^]^.

Initial management often include bony stabilization and surgical debridement of non-viable tissues. Herein, bony stabilization was done using K-wires, which was the most widely used implants in these scenarios^[^10^]^. Any spacer, such as bone cement, was not used at the bony defect because of medial sided skin and soft tissue loss. Preservation of skin was attempted during debridement as much as possible. Broad-spectrum antibiotics (3rd generation cephalosporin and gentamicin) were initiated at admission, and tissue samples were sent for culture and sensitivity. The culture revealed *Klebsiella* species resistant to the initial antibiotic. This contributed to the progression of localized infection, necrosis, and skin loss. Earlier identification of the resistant organism and the use of adjunctive therapies such as hyperbaric oxygen therapy and vacuum-assisted wound closure in such cases could have potentially limited tissue damage^[^11,12^]^. However, as we did not have access to either hyperbaric oxygen therapy or vacuum-assisted closure devices, we opted for surgical debridement on day 4. Once sensitivity results were available, antibiotic therapy was escalated to piperacillin-tazobactam. A 5th ray amputation and debridement of necrotic tissue were performed.

Localized infection was controlled and plastic surgery team was consulted for soft-tissue reconstruction, as the defect required free-flap coverage.

The term “orthoplastic approach” has been a commonly used term in cases where orthopedic surgery and plastic or reconstructive surgery team gets involved^[^13^]^. Although varieties of reconstructive procedures, such as skin grafting, supra-malleolar pedicle flap, or reverse sural flap, has been performed by orthopedic surgeons, this case particularly required free flap, as the defect lied over dorsal and medial aspect of the foot with exposed tendons and bones^[^14–16^]^.

A single stage reconstruction using vascularized osteocutaneous flap was a valid option^[^17,18^]^. However, plastic surgery team opted for chimeric flap, i.e., anterolateral thigh free flap with vastus lateralis muscle pedicle for the reconstruction^[^19^]^.

This combination has provided maximum freedom for reconstruction. Dorsal defect was reconstructed using fasciocutaneous component and muscle pedicle was used to reconstruct medial defect which was covered with split thickness skin graft. This combination flap also had an advantage of low donor site morbidity, as the donor site was closed primarily. Some previous reports have also shown excellent results of foot and ankle defect reconstruction using these flaps^[^20^]^.

Reconstruction of the bony defect at medial arch, which was extended from the base of 1st meta-tarsal to navicular, was also challenging. An antibiotic bone cement spacer is usually placed in these defects, if planned for later stage grafting, to help formation of pseudo membrane as well as local delivery of antibiotics^[^21^]^.

However, it was not opted in this case because of patient’s choice. In this case, a tricortical iliac crest graft was harvested and interposed into the defect and stabilized using titanium mini-plates and screws. PubMed database search using key words “Tricortical iliac crest graft” resulted in limited number of reports regarding the use of tricortical iliac crest graft for traumatic foot defect reconstruction. Some previous reports have shown that the tricortical iliac crest bone graft can successfully be used to reconstruct mid-foot bony defects caused by en bloc resection of tumors and to perform small joint arthrodesis^[^22–24^]^. The decision to use a tricortical iliac crest bone graft was based on its superior structural and biological properties. Tricortical grafts provide robust cortical strength and cancellous content, allowing for both mechanical support and osteoconductive potential, which are essential for midfoot reconstruction where load-bearing forces are significant^[^25^]^. Studies have shown that tricortical iliac crest grafts maintain their shape and volume better than cancellous grafts alone, reducing the risk of collapse and non-union in arthrodesis procedures. Compared to allografts or synthetic substitutes, autologous tricortical grafts offer enhanced biological integration, reduced immunologic response, and better long-term outcomes^[^26^]^. Their application has been well documented in complex reconstructions of the midfoot and hindfoot, especially in cases of trauma, tumor resection, or infection where structural voids need stable bridging.

The patient was followed up for 18 months, and at that time, he was walking with mild discomfort due to the complexity of the injury and reconstruction. However, he was very satisfied with the outcome and able to perform activities of daily living. The graft healed and ankle range of motion was 15° dorsiflexion and 30° plantarflexion. Restricted range of motion could be due to soft-tissue scarring around the joint, loss of muscle belly during debridement, and longer period of immobilization. AOFAS scale is a validated tool for the evaluation of foot and ankle function^[^27^]^. In this case, the patient had AOFAS scale of 80, which is regarded as good functional recovery.

### Conclusion

Early reconstruction of complex foot injury using orthoplastic approach may lead to survival of the limb and satisfactory functional recovery. However, longer follow-up is required to assess the occurrence of post-traumatic arthritis of mid-foot and ankle joints.

## Data Availability

Data Sharing will be publicly available.

## Research registration unique identifying number (UIN)

Not applicable for case reports.
